# Effect of heat–moisture treatment on digestibility of different cultivars of sweet potato (*Ipomea batatas* (L.) Lam) starch

**DOI:** 10.1002/fsn3.115

**Published:** 2014-04-20

**Authors:** Suraji Senanayake, Anil Gunaratne, K K D S Ranaweera, Arthur Bamunuarachchi

**Affiliations:** 1 Department of Food Science & Technology, University of Sri JayewardenepuraColombo, Sri Lanka; 2 Faculty of Agricultural Sciences, Sabaragamuwa University of Sri LankaBelihuloya, Sri Lanka

**Keywords:** Digestibility, heat–moisture treatment, sweet potato starch

## Abstract

Different heat–moisture levels were applied to native starches from different cultivars of sweet potatoes available in Sri Lanka (Wariyapola red, Wariyapola white, Pallepola variety, Malaysian variety and CARI 273) to study the digestibility level. Samples were treated with 20, 25, and 30% moisture at 85°C and 120°C for 6 h and in vitro starch digestibility was tested with porcine pancreatin enzyme. A range of 19.3–23.5% digestibility was shown by the native starches with no significant difference (*P* < 0.05). Significant changes were observed in the digestibility level of the hydrothermally modified starches and the moisture content showed a positive impact on the digestibility. Heat–moisture treatment at 85°C brought an overall increase in digestibility and temperature beyond 85°C had a negative impact. No significant difference (*P* < 0.05) in the digestibility was observed with 20% and 25% moisture at 85°C and increased level were seen at 85°C and 30% moisture.

## Introduction

The production of sweet potatoes (*Ipomea batatas* (L.) Lam) in the tropics and subtropics has increased significantly over the recent years and it is the seventh most important food crop in the world (Horton 1988). In many developing countries sweet potatoes are being used as supplementary staple food and animal feed. A major constraint in the application of sweet potatoes in animal feed is its poor starch digestibility (Yeh and Bouwkamp 1985). Dreher et al. (1984) found that uncooked sweet potato starch was less susceptible to amylolysis compared to uncooked cereal starches. The amount of digestibility or amylolysis depends on the chemical structure of the starch, physical arrangement, the presence of possible inhibitors, and the physical distribution of starch in relation to dietary fiber components such as cellulose, hemicelluloses, and lignin (Snow and O'Dea 1981).

In order to understand the nutritive value and to apply in various industries it is important to understand the digestibility of starch by enzymes. Delpeuch and Favier (1980) reported that sweet potato starch was found to be more susceptible to enzyme degradation by *α*-amylase and glycoamylase than the cassava starch. Starch digestibility can be measured by using simple in vitro systems; however, in vivo digestibility studies show more complete amylolysis studies. Precooking efficiently increases the starch digestibility and thereby increases the feeding efficiency for human consumption but this process is not as economical for use as animal feed. The increase in starch digestibility on cooking can be related to the changes that occur in the starch structure due to gelatinization (Moorthy and Padmaja 1991). Significant differences in starch digestibility in different varieties were reported by Tsou and Hong (1989) and Madamaba et al. (1975). A significant cultivar variation in starch digestibility of sweet potatoes was also reported by Zhang and Oates (1999). Variation in starch digestibility was found to occur according to the climatic condition of the growing season and developmental stage of the plant (Tsou and Hong 1989; Noda et al. 1996).

Native starches can be modified to increase its digestibility by physical or chemical means. Heat–moisture treatments (HMTs) can alter the structure of the starch granules and thereby change the susceptibility to enzymatic attack. Hydrothermal treatments to starch involve only heat and moisture, thus these modifications are considered to be natural and safe (Jacobs and Delcour 1998).Two basic types of HMTs are commonly employed in modifying the physicochemical properties of starch (Stute 1992). One method is “annealing” (Knutson 1990), which involves treating the starch with excess levels of moisture and the other method is to add restricted levels of moisture for the treatment of starch which is referred to as “HMT” of starch (Hoover and Vasanthan 1994; Collado and Corke 1999).

Depending on the conditions applied to HMT treatment, starch solubility, gelatinization temperature, enzymatic susceptibility, and changes in the X-ray diffraction patterns occur (Donovan et al. 1983; Abraham 1993). No comprehensive research has been carried out on the digestibility changes in sweet potato starch with different levels of HMT. Hence, this study was carried out to investigate the effect of varying the levels of HMTs on starch digestibility in five different cultivars of sweet potatoes popular in Sri Lanka.

## Material and Methods

From Dhambulla, Gokarella, and Horana areas in Sri Lanka mature tubers of sweet potato cultivars namely, swp 1 (Wariyapola red), swp 3 (Wariyapola white), swp 4 (Pallepola variety), swp 5 (Malaysian variety), and swp 7 (CARI 273) were randomly selected. Samples were collected from market areas and the cultivars were identified at the Horticultural Crop Research and Development Institute, Gannoruwa, Kandy, Sri Lanka. Flour and starch samples were prepared 2–3 days after harvesting.

### Starch extraction

Starch separation was carried out according to the method described by Takeda et al. (1988) with slight modifications. Fresh tubers were washed, peeled, and diced. These cubes were then dipped in ice water containing 100 ppm sodium metabisulphite to minimize browning and were wet milled at low speed in a laboratory scale blender with 1:2 w/v of tap water for 2 min and filtered through a gauze cloth. Residue was repeatedly wet milled, filtered thrice, and the suspension was kept overnight for settling of starch. The supernatant was decanted and the settled residue was further purified with repeated suspension in tap water (1:2 v/v) followed by settling for 3 h. The purified starch was dried at 35°C, sifted through 300 *μ*m sieve, sealed, and packed for analysis.

### Heat–moisture treatment of the extracted starch

From the extracted starch, 20 g of each sample was taken and the moisture levels were adjusted to 20, 25, and 30%. The moisture-adjusted samples were placed in tubes with a sealing cap and equilibrated at room temperature for 12 h. Samples were heated at 85°C and 120°C for 6 h. Occasional shaking was done to the samples within the treatment period for homogeneous distribution of moisture. After treatment, the samples were cooled to room temperature and dried at 40°C to a uniform moisture level of 10% and equilibrated at room temperature for 2 days.

### Digestibility of native and heat–moisture-treated starch

Starch digestibility was measured by the method described by Senanayake et al. (2013). A sample of 500 mg was placed in a weighed centrifuge tube (Tarsons, 50 mL) with addition of 15 mL phosphate buffer (0.15 mol/L, pH 6.5), 30 mg CaCl_2,_ 30 mg gelatin, and 30 mg pancreatin (Sigma Co., St. Louis, MO). The capped tubes were placed in a shaking water bath at 37°C with sufficient speed to keep the flour in suspension for 12 h and the reaction was stopped with the addition of 5 mL of 1% H_2_SO_4_. The suspension was centrifuged at 20,000*g* for 10 min and the supernatant was decanted and the residue pellet was dispersed with 15 mL of 80% ethanol and recentrifuged for 5 min. The supernatant was decanted and the tubes with the residue pellet were dried at 50°C for 6 h, then at 80°C to constant weight, cooled, and weighed. Starch digestibility was expressed as percent weight loss after digestion. A blank without pancreatin was included for each sample to adjust the results.

### Statistical analysis

Native starches containing 10% moisture and ambient temperature of 30°C were treated with different levels of heat and moisture for 6 h. The experiment was a three-factor factorial design. The results were subjected to General Linear Model and Analysis of variance and correlation analysis for this study were carried out by using MINITAB version 14 (Portland, OR).

## Results and Discussion

The digestibility values of native starches from the five different cultivars of sweet potatoes were in the range of 19.3–23.5% and there was no significant difference (*P* > 0.05) under the untreated condition (Table 1). Flour digestibility studies done on these cultivars with pancreatin enzyme was in the range of 36–55% and the lowest and the highest values were found in swp1 and swp7 cultivars, respectively (Senanayake et al. 2013). Pancreatin contains the pancreatic enzymes, trypsin, amylase, and lipase, thus percentage starch digestibility due to pancreatic amylase in native starch would be somewhat lower compared to the digestibility of the flour. Zhang and Oates (1999) reported a significant level of variation in starch digestibility in different cultivars of sweet potatoes. Although they observed a strong positive correlation of starch digestibility with flour digestibility there was no positive correlation found with flour and starch digestibility in this study. Woolfe (1992) and Rasper (1969) reported that that raw starch had poor digestibility around 24% with *α*–amylase enzyme, which was in agreement with our findings.

**Table 1:** In vitro starch digestibility^1^ of native starches of different sweet potatoes

Sweet potato cultivar	Digestibility
swp 1 (Wariyapola red)	21.7 ± 0.7^a^
swp 3 (Wariyapola white)	21.9 ± 1.5^a^
swp 4 (Pallepola variety)	23.5 ± 0.9^a^
swp 5 (Malaysian variety)	23.5 ± 0.4^a^
swp 7 (CARI 273)	19.3 ± 0.3^a^

Values given with the same superscript do not have a significant difference at *P >* 0.05 level.

1 Digestibility was expressed as percentage weight loss after digestion with pancreatin.

Digestibility values of all the starches changed after HMT, but the extent and trends of changes were different under different HMT conditions. Previous studies showed early swelling of starch granules due to HMT treatments and the effects of HMT on digestibility showed marked moisture content dependence (Table 2). And from the analysis of mean values for digestibility in different cultivars, there was a clear increase in digestibility with more than 20% moisture content in all the cultivars, except swp7. But it is evident from the results that the gradual increase in moisture level enhanced the level of digestibility.

**Table 2:** Changes in in vitro starch digestibility^1^ of sweet potato starches after HMT^2^ under different conditions

Temperature (°C)	Moisture (%)	swp1	swp3	swp4	swp5	swp7
85	20	13.2 ± 0.3^d^	23.5 ± 0.9^b^	26.6 ± 0.7^a^	19.5 ± 0.5^c^	13.7 ± 0.2^d^
	25	32.2 ± 1.2^b^	28.8 ± 0.4^c^	37.6 ± 0.5^a^	29.2 ± 0.4^c^	16.4 ± 0.4^d^
	30	44.1 ± 2^b^	42.9 ± 0.4^b^	43.3 ± 0.3^b^	53.0 ± 0.1^a^	27.2 ± 0.3^c^
120	20	12.2 ± 0.2^b^	9.4 ± 0.5^c^	15.2 ± 0.3^a^	4.9 ± 0.2^d^	3.9 ± 0.2^d^
	25	23.1 ± 0.4^b^	20.3 ± 0.3^c^	20.1 ± 0.3^c^	29.4 ± 0.4^a^	10.9 ± 0.1^d^
	30	35.6 ± 0.6^a^	24.6 ± 0.5^c^	33.4 ± 0.6^b^	34.4 ± 0.3^b^	13.3 ± 0.2^d^

Values represented by similar superscripts in each row are not significantly different at *P* > 0.05. HMT, heat–moisture treatment.

1 In vitro digestibility was expressed as percent weight loss after digestion.

2 HMT time is 6 h.

Heat–moisture treatment at 85°C brought about an overall increase in digestibility. Although there were different extents of change, all starches showed progressive increase in digestibility with 30% moisture content (Table 2). Franco et al. (1995) found that there was an increased level of enzyme susceptibility for normal starches, with moisture levels more than 18%. Hoover and Vasanthan (1994) reported that HMT at moisture 30% at 100°C for 16 h increased the enzymatic hydrolysis of oat, potato, and yam starches. The mean values of digestibility obtained for different levels of HMTs for cultivars were significantly different at *P* < 0.05 level. Comparably high level of digestibility was apparent when the starch was treated with 30% moisture at 85°C for 6 h than the other treatments (Table 2). Significantly low level (*P* > 0.05) of digestibility was observed in samples treated with 20% moisture at 120°C and these values were even lower than the values obtained for the native starches of each cultivar (Tables 1, 2). Significantly high level of digestibility than the native starch was observed in the treatment levels of 25% moisture at 85°C and 30% moisture at 120°C.

The changes produced by heating starches at or below 100°C under high moisture contents are physical and these physical changes result from an increased degree of association of starch molecules within the granule. The moisture content of the starch is an important factor in effecting this physical change since the water within the granule apparently permits the starch molecules or a part of them to rotate. This brings out the intimate contact within the chains of starch molecules and creates an increased number of hydrogen bonds. This may bring about the rearrangement of the molecules within the granules resulting in changed X-ray diffraction pattern. Low levels of moisture at high temperature levels induce the physical reorganization within granules (Stute 1992; Franco et al. 1995; Tester and Debon 2000).

Negative correlation was observed in digestibility beyond 85°C and this holds especially true in the case of swp7 cultivar, where the values were even lower than the native starch (Table 2). Therefore, we can assume that newly created molecular structure changes within the starch granule due to high-heat treatment alone, may be less susceptible to enzymatic attack than its native structure. The values are even lower at low moisture level and high-heat treatment (Table 2). Study of X-ray diffraction pattern of native and HMT starch of these cultivars is important in explaining these changes. The newly created molecular structures at 120°C temperature in these cultivars are more prone to act as resistant starch toward enzymes. Formation of resistant starch with repeated HMT treatments in potato and high-amylase maize starches was observed by Kobayashi (1993).

**Figure 1:**
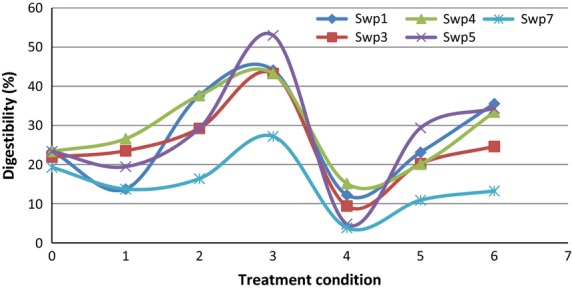
Effect on in vitro starch digestibility with different levels of heat–moisture treatment for 6 h.

Enzymes erode the entire starch granular surface (exocorrosion) or digestion proceeds through selected channels on the granule surface toward the center (endocorrosion). Starches from sweet potatoes are found to be having specific susceptible zones to *α*-amylase (Oates 1997). The regions that are susceptible to enzyme attack are mostly the amorphous regions of the granule. Therefore, the alteration of the crystalline structure of the granule by using heat and moisture can readily influence the susceptibility for enzymatic degradation. Our results show that the higher temperature of 120°C and low moisture level of 20% might have caused rearrangement of starch molecular structure and strengthened the forces which maintained the granules. This resulted in reducing the number of channels on the granular surface which make way to the enzyme attack of the interior. This was clearly evident in swp7 cultivar and the incidents of occurring more number of channels due to molecular rearrangement, can take place at higher moisture levels and slightly higher temperatures than their gelatinization temperatures. Our results clearly show that in all the cultivars we studied at 120°C and higher moisture levels (25% and 30%), digestibility was significantly higher (*P* < 0.05) than at 120°C and 20% moisture level (Fig. 1).

The results clearly show that the moisture levels between 25–30% and 85°C HMT resulted in rupture or weakening of the forces that maintain the granules thus creating more accessible areas for enzymatic attack. Fresh tubers of sweet potatoes are commonly eaten boiled or as a curry with other food ingredients. Heat processing with high moisture levels can increase the level of starch digestibility when exposed to salivary and pancreatic amylase in human consumption. Application of sweet potato in animal feed should be done after HMT in order to increase the nutritive index of the feed. Therefore, economic ways of improving the digestibility of these starches should be further analyzed if we intend to include these sweet potato cultivars in animal feed substitutions.

## Conclusion

There was no significant difference (*P* < 0.05) in the digestibility level of the native starches. All starches showed significant changes in the extent of digestibility with different levels of HMT. Moisture level in the treated starch had a strong positive correlation with digestibility, while temperature increases beyond 85°C with low moisture levels below 20% had a negative impact on the digestibility. All starches showed high levels of digestibility with 30% moisture content and highest digestibility was observed at 85°C and 30% moisture level in all cultivars (swp5 being the highest and swp7 being the lowest). It can be assumed that the extent of molecular rearrangement due to levels of HMT and the resulting changes in crystalline structure bring about varying levels of digestibility, due to the changes occur for the enzymatic exposure.
